# Total saponins from *Trillium tschonoskii* Maxim promote neurological recovery in model rats with post-stroke cognitive impairment

**DOI:** 10.3389/fphar.2023.1255560

**Published:** 2023-09-07

**Authors:** Gang Wang, Xiane Tang, Fangyu Zhao, Xiaoli Qin, Fengjie Wang, Dan Yang, Hong Zhu, Xianbing Chen

**Affiliations:** ^1^ Hubei Provincial Clinical Medical Research Center for Nephropathy, Minda Hospital of Hubei Minzu University, Enshi, China; ^2^ Health Science Center, Hubei Minzu University, Enshi, China

**Keywords:** total saponins from *Trillium tschonoskii* Maxim, post-stroke cognitive impairment, neuronal synaptic, apoptosis, Shh signaling pathway

## Abstract

Total saponins from *Trillium tschonoskii* Maxim (TSTT), a bioactive component of local natural herbs in the Enshi area, China, have been demonstrated to have functions of restoring cognitive capacity and promoting axonal regeneration post-stroke, but the mechanism of this process remains unclear. The hippocampus is a critical tissue for controlling learning and memory capacity, and the sonic hedgehog (Shh) signaling pathway plays a major role in the patterning and synaptic plasticity of hippocampal neural circuits. Therefore, we aimed to investigate whether TSTT could restore learning and cognitive functions by modulating the Shh pathway in rats with post-stroke cognitive impairment (PSCI). The ischemia model was established by permanent middle cerebral artery occlusion (MCAO) in 100 Sprague–Dawley (SD) rats, and the model rats were administered using TSTT (100 mg/kg) or donepezil hydrochloride as the positive control (daily 0.45 mg/kg, DON) for 4 weeks after the operation. As assessed by the Morris water maze test, the cognitive function of PSCI rats was significantly improved upon TSTT treatment. Meanwhile, the cerebral infarct volume reduced with TSTT, as shown by HE and TTC staining, and the number of Nissl bodies and dendritic spine density were significantly increased, as shown by Nissl and Golgi staining. In addition, TSTT upregulated PSD-95, SYN, and GAP-43, and inhibited neuronal apoptosis, as evidenced by increased Bcl-2 levels along with decreased Bax and caspase-3 expression. TSTT could also significantly upregulate Shh, Ptch1, Smo, and Gli1 proteins, indicating the activation of the Shh signaling pathway. Therefore, TSTT can protect PSCI rats by inhibiting apoptosis and promoting neuronal synaptic remodeling. The Shh pathway is also involved.

## Graphical Abstract

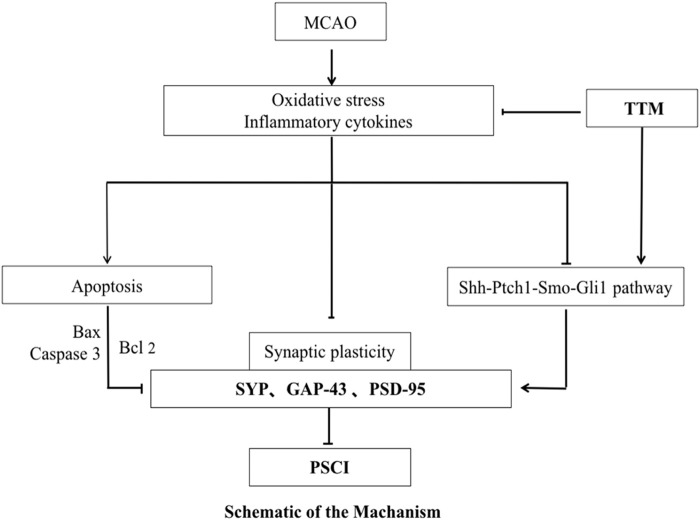

## 1 Introduction

Post-stroke cognitive impairment (PSCI) ranges from mild cognitive impairment to stroke-related dementia (Zhang and Bi, 2020). The HE prevalence of stroke in China in 2019 was 2022/100,000, the incidence of stroke was 276.7/100,000, and the mortality was 153.9/100,000 (Ma et al., 2021). In addition, PSCI can occur in over 1/3 of stroke patients, but the mechanism of PSCI remains unclear (Kim et al., 2020). PSCI is characterized by chronic progressive cognitive impairment. It is a significant drain on medical resources and affects the quality of life of patients.

*Trillium tschonoskii* Maxim (TTM) is a traditional Chinese herb used to treat traumatic brain injury and headache. Diosgenin glucoside is the bioactive component of total saponins from *Trillium Tschonoskii* Maxim (TSTT) that has a clear neuroprotective effect, but its pharmacological mechanism is still unclear. Previous studies have confirmed that TSTT can reduce spinal cord injury and promote spinal nerve regeneration by inducing autophagy through downregulation of miR-155 and activation of the Rheb/mTOR signaling pathway (Chen et al., 2018). Sequential studies have demonstrated that TSTT can induce autophagy by activating the Rheb/mTOR signaling pathway, thereby alleviating learning and memory impairment in D-galactose-induced aging rats and exerting anti-aging effects (Wang et al., 2018). Currently, TSTT has been widely used for ischemic stroke and other neurodegenerative diseases. However, the neuroprotective mechanism of TSTT remains to be elucidated.

The sonic hedgehog (Shh) pathway consists of Shh ligands, receptor complexes composed of the transmembrane receptors Patched1 (Ptch1) and Smoothened (Smo), and Gli1. When the ligand Shh binds to Ptch1, the inhibitory state of Ptch1 on Smo is released, and Smo is dissociated and translocated from the cytoplasm to the primary cilia, thereby triggering Gli1 to enter the nucleus and inducing the transcription of downstream genes (Katoh, 2019). Shh signaling engages in embryonic development, stem cell proliferation and differentiation, axon growth, synapse formation, and angiogenesis, and is critical for the development of the central nervous system (Patel et al., 2017; Hoyos Cadavid et al., 2019; Katoh, 2019). The endogenous Shh signaling pathway is activated in pathologies like ischemic stroke, brain trauma, and infection, thereby suppressing brain tissue damage and promoting neurogenesis and synaptic remodeling (Alvarez-Buylla and Ihrie, 2014; Zhang et al., 2014; He et al., 2017). Activating the Shh signaling pathway may elevate neuronal plasticity and improve cell survival (Ding et al., 2013).

Previous research studies exhibit that the activated Shh signaling pathway in animal models of stroke can improve brain plasticity, reduce apoptosis, and promote angiogenesis. Similarly, the Shh signal pathway can also enhance synaptic plasticity and synaptic connections, thereby alleviating cognitive dysfunction (Chen et al., 2002; Ung et al., 2018). Exogenous application of Shh peptides or Shh signaling pathway agonists can significantly increase the micro-vessel density and neuronal survival rates in the cerebral ischemic boundary area of wounded mice, thereby rescuing cognitive impairment, indicating that the Shh signaling pathway plays a major protective role in response to ischemia. Furthermore, in the adult hippocampus, the Shh signaling receptors Ptch1 and Smo are expressed in neuronal dendrites, and local activation of Smo activates Gli1, inducing axonal actin-binding protein expression, promoting the growth of neuronal axons, and accelerating their interaction with the dendrites of target neurons and synaptic connections (Jin et al., 2015).

Given the multifaceted capacity of the Shh signaling pathway in regulating brain function reconstruction, we speculate that TSTT plays a beneficial role in nerve repair in PSCI model rats through the Shh signaling pathway.

## 2 Materials and methods

### 2.1 Animals

A total of 100 healthy male Sprague–Dawley (SD) rats, with the age of 10 weeks and body weight of 200–220 g, were purchased from Hunan Slake Jingda Experimental Animal Co., Ltd. (Certificate No: SCXK(X)2019-0004). The experimental protocols were approved by the Institutional Animal Care and Use Committee from Hubei Minzu University, China.

### 2.2 Experimental procedures

All rats in the model group were treated by a modified Zea–Longa suture method for middle cerebral artery occlusion (MCAO) to establish a rat model with transient left cerebral ischemia–reperfusion injury. The specific operation was performed as follows: before surgery, all rats were fasted for 12 h without water, anesthetized with 2% pentobarbital sodium (2 mL/kg) by intraperitoneal injection, and prepared for skin disinfection; the subcutaneous tissue was bluntly dissected, exposing the common carotid, internal carotid, external carotid, and pterygopalatine arteries. The proximal ends of the common carotid and external carotid arteries were ligated, and the internal carotid artery was clipped. The common carotid artery was cut using scissors to form a V-shaped opening, and a suture was inserted at a depth of approximately 18.5 mm. The living knot was ligated, sutured layer by layer, and reperfused for 2 h. The Sham surgery group had the same operation as the model group, but only the skin was cut, the left common carotid artery was separated, and the incision was sutured without embolization.

The rats in the model group were screened by the Zea–Longa score and randomly divided into three groups, including the TSTT group with a daily intragastric administration of 100 mg/kg for 28 days, donepezil hydrochloride as the positive control group (DON) with a daily intragastric administration of 0.45 mg/kg for 28 days, and the model group (MCAO) administered with the same volume of normal saline every day, as well as the sham operation group (Sham) administered with the same volume of normal saline every day. Eighteen rats in each group were subjected to the water maze behavior test on the day 21 of intragastric administration. All rats were euthanized after the behavioral test. Three rats in each group were randomly selected for anesthesia, normal saline was perfused into the heart, and 400 mL of neutral paraformaldehyde was perfused for 60 min. Another three rats were randomly selected for TTC staining, and hippocampal tissues were harvested from the remaining rats after cardiac perfusion with normal saline and stored in a refrigerator at −80°C for later use.

### 2.3 Behavioral assessment

The Morris water maze pool was 160 cm in diameter, 80 cm in height, and 22°C ± 2°C in water temperature. Prior to the experiment, the rats in each group were placed in the water maze laboratory to adapt to the environment. The water maze experiment included a positioning navigation experiment and a space exploration experiment. 1) The positioning navigation experiment lasted for 5 days. At the beginning of the experiment, the rats were slowly put into the pool from the first, second, and fourth quadrants. The swimming trajectory of the rats within 60 s was systematically recorded, and the time to find the survival platform within 60 s was the escape latency. 2) In the space exploration experiment, the platform was removed and the rats were put into the first quadrant in order. The time required for the rats to swim through the original position of the platform for the first time was within 60 s, and the number of rats crossing the platform and the residence time spent in the third quadrant were recorded, counted, and analyzed.

### 2.4 TTC, HE, and Nissl staining

TTC staining was performed twice: the first time on day 7 of intragastric administration and the second time at the end of the behavioral evaluation. The specific operation was performed as follows: fresh brain tissue was frozen at −20°C for 20 min, cut into seven pieces of 2 mm thickness along the coronal position, and then placed in a culture dish with 2% TTC staining solution in a 37°C incubator for 15–30 min. After washing with normal saline, the slices were fixed with 10% paraformaldehyde solution for 1 h and photographed. Image-Pro Plus 6.0 software was used to measure the volume ratio of the ischemic area in each group. The volume ratio of the ischemic area was calculated using the following formula: (the sum of the white ischemic area of each slice − the sum of the error white area of the Sham group) / (the sum of the brain slice area of each slice) × 100%. The fixed brain tissue was dehydrated, embedded in paraffin, and then sliced at 4 μm thicknesses; after dewaxing and hydration, the paraffin sections were subjected to HE and Nissl staining, and the neurons and Nissl bodies in the hippocampal CA1 region were observed using an optical microscope. Image-Pro Plus 6.0 software was used to count the volume of the ischemic area and the area of Nissl bodies in each group.

### 2.5 TUNEL and Golgi staining

The 4-μm sections of brain tissue were dewaxed, hydrated, and stained, according to the instructions of the TUNEL staining kit. Sections were incubated in the DAPI staining solution for 3 min and observed using an optical microscope. The fresh hippocampal tissue from the left ischemic lesion area was cut into 5-mm-thick pieces, and the brain tissue was fixed for 2 weeks, according to the instructions of the Golgi staining kit. The tissue was cut into 50-μm-thick sections. The shape and density of dendritic spines in ischemic neurons were observed using a microscope after staining. The apoptotic rate of neurons and the density of dendritic spines in each group were analyzed by Image-Pro Plus 6.0 software.

### 2.6 Immunohistochemical fluorescence

The brain tissue wax block was cut into 4-μm slices, dewaxed, hydrated, and antigen-repaired. After incubation with 3% BSA for 30 min, the primary antibody was added dropwise and incubated overnight at 4°C. After rewarming, PBS was washed three times for 3 min each time, then the secondary antibody was added and incubated at room temperature for 50 min, and DAPI was incubated at room temperature for 10 min. Images were captured using a fluorescence microscope.

### 2.7 Protein analysis by Western blot

An appropriate amount of hippocampal tissue was weighed, and the suspension of lysate and PMSF was added according to the ratio of tissue weight (mg): lysate volume (μL): PMSF volume (μL) at 10:100:1. The supernatant was obtained by ice-cracking, homogenization, and centrifugation. The protein concentration was determined using the BCA method. The protein was denatured at 100°C for 5 min, separated by SDS-PAGE, transferred to the PVDF membrane at 250 mA, blocked with the rapid blocking solution for 15 min, and washed three times with TBST for 10 min each time. The primary antibody was incubated overnight at 4°C, the membrane was washed three times with TBST, and the secondary antibody was incubated for 1 h at room temperature. The LAS4000 gel imaging system was used to process, collect, and analyze the target protein.

### 2.8 Statistical analysis

SPSS 22.0 software was used for statistical analysis. Experimental data were expressed as the mean ± standard error (M ± SE). One-way ANOVA was used for comparison between groups, and the LSD test was used for comparison between groups. The statistically significant difference was considered at *p* < 0.05. A histogram was plotted using GraphPad Prism 8.0 software.

## 3 Results

### 3.1 TSTT improved cognitive dysfunction in rats with PSCI

**FIGURE 1:**
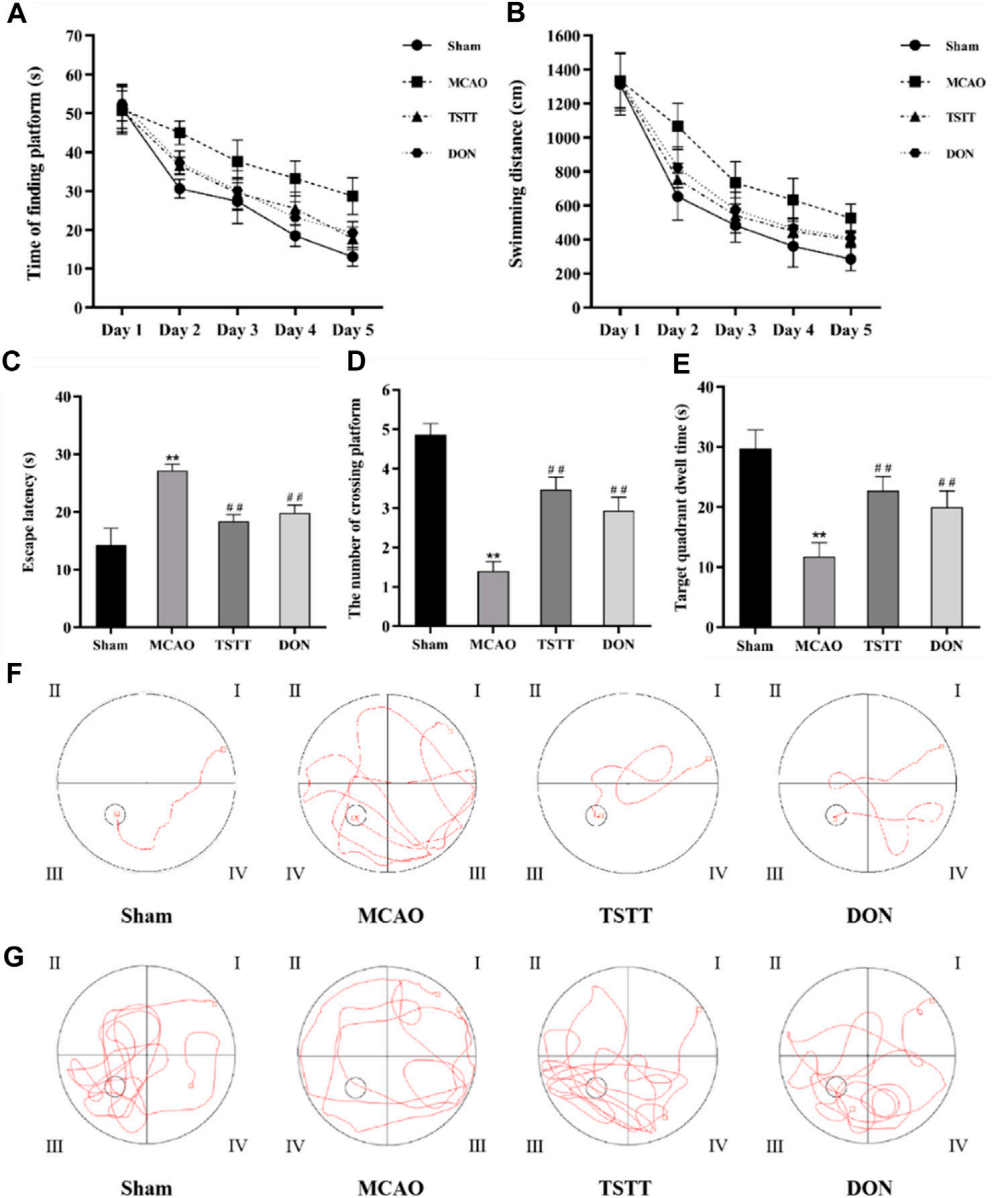
Morris water maze test results and representative swimming trajectory of rats from each group: **(A)** time to find the platform, **(B)** swimming distance, **(C)** escape latency, **(D)** the number of crossing platforms, **(E)** target quadrant dwell time, **(F)** swimming trajectory map for positioning navigation experiments, and **(G)** swimming trajectory map for space exploration experiments. ^**^ *p* < 0.01 vs. Sham group; ^##^ *p* < 0.01 vs. MCAO group; *n* = 15.

To determine whether TTM can contribute to the improvement of cognitive dysfunction in PSCI rats, the Morris water maze test was performed to evaluate spatial learning and memory capacity. On day 1, there was no significant difference between groups in the time to find the platform or swimming distance; however, the rats in the MCAO group spent significantly longer time to find the platform than those from the Sham group on each day of the training phase (Figure 1A, *p* < 0.01). The total swimming distance (Figure 1B) and the latency to find the platform (Figure 1C) were also prolonged in the MCAO group (*p* < 0.01). Conversely, the number of rats crossing the platform (Figure 1D) and target quadrant dwell time (Figure 1E) were significantly reduced in the MCAO group when compared with the Sham group (*p* < 0.01). Additionally, all these phenomena can be reversed by TSTT and DON treatments (*p* < 0.01). These data demonstrated that the rats from the MCAO group had impaired spatial memory, and TSTT could significantly enhance the spatial learning and memory capacity of rats with PSCI.

### 3.2 TSTT reduced the infarct volume after cerebral ischemia/reperfusion

**FIGURE 2:**
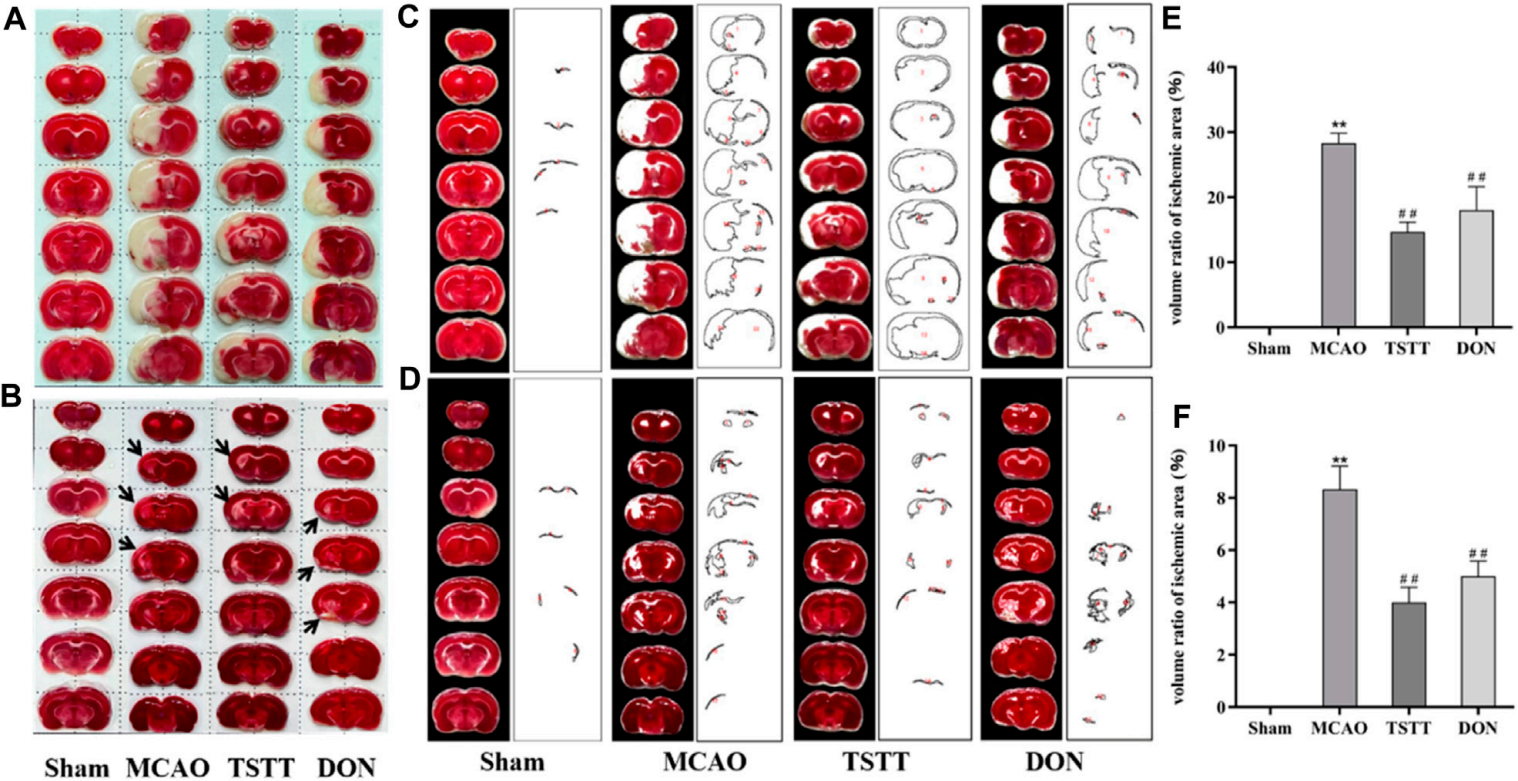
TTC staining and statistical results of brain tissue in rats from each group. **(A)** TTC staining after intragastric administration for 7 days. **(B)** TTC staining after intragastric administration for 28 days. **(C)** Quantification of the ischemic volume after intragastric administration for 7 days. **(D)** Quantification of the ischemic volume after intragastric administration for 28 days. **(E)** Comparison of the ischemic volume after 7 days of intragastric administration. **(F)** Comparison of the ischemic volume after 28 days of intragastric administration. ^**^ *p* < 0.01 vs. Sham group; ^##^ *p* < 0.01 vs. MCAO group; *n* = 3.

To understand the potential mechanisms of TTM for promoting cognitive function, we observed the morphological alterations of brain tissue in PSCI rats. TTC staining was performed after 7 and 28 days of intragastric administration. As shown in Figures 2A–F, compared with the MCAO group, TSTT treatment reduced the infarct volume significantly (p < 0.01).

### 3.3 TSTT promoted neuronal function and survival in the ischemic penumbra

**FIGURE 3:**
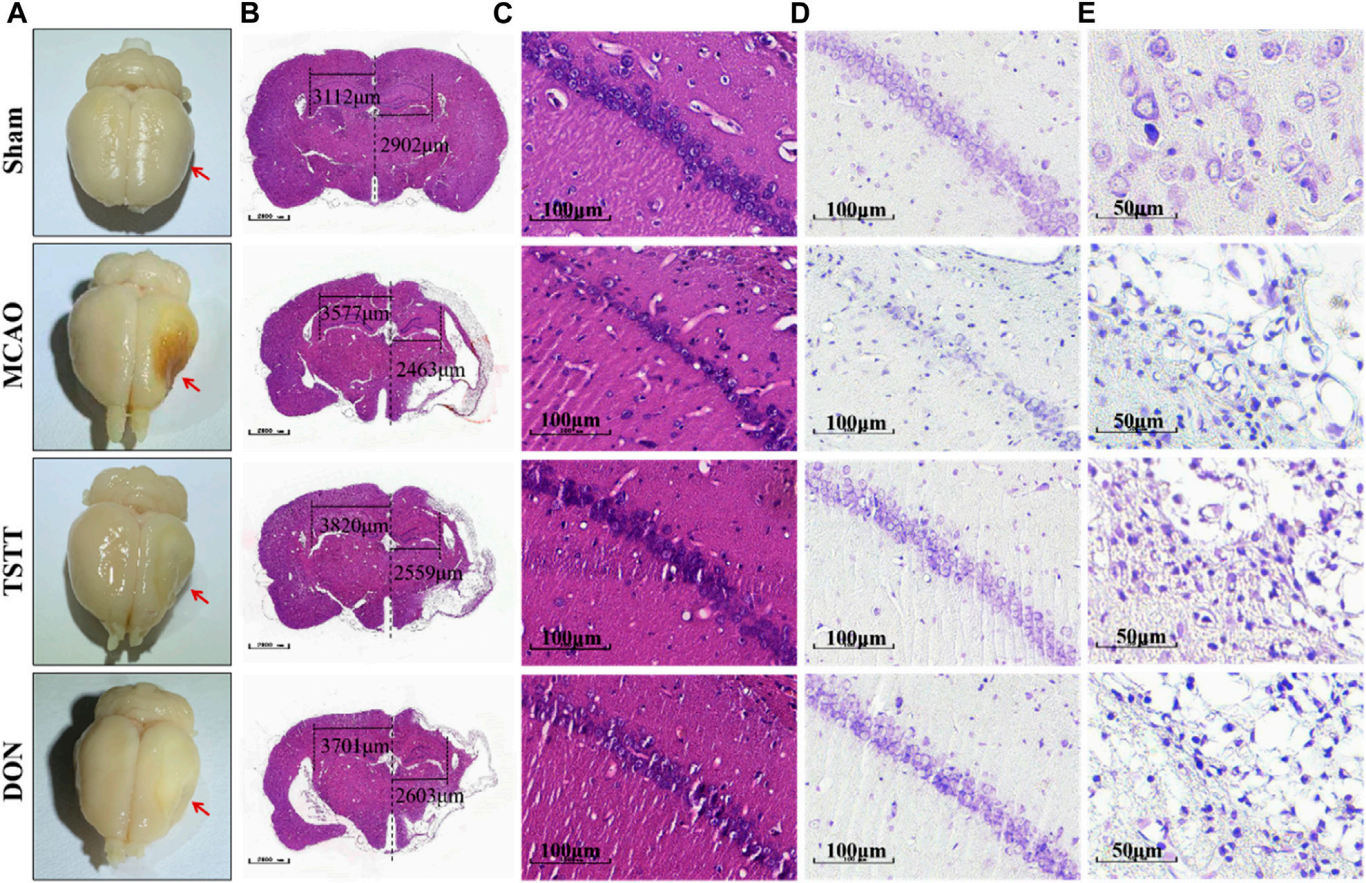
HE and Nissl staining after brain tissue perfusion in each group:**(A)** Representative map of brain tissue perfusion in each group. **(B)** HE staining of whole brain tissue; Scale bar = 1,000 μm. **(C)** HE staining of the hippocampal CA1 region; scale bar = 100 μm. **(D)** Nissl staining of the hippocampal CA1 region a; scale bar = 100 μm. **(E)** Nissl staining in the cortex and ischemic penumbra; scale bar = 50 μm.

HE and Nissl staining were performed for histopathological analysis to assess the degree of neuronal cell death. After perfusion fixation of brain tissue, we observed different degrees of atrophy of the brain tissue in rats from each group (Figure 3A). HE staining suggested that MCAO rats exhibited histological changes when compared with the Sham group, which was attenuated by TSTT in the CA1 region of hippocampal tissues in rats (Figures 3B,C). Nissl staining showed that the density and number of healthy neurons in the CA1 areas of the hippocampus in rats from the MCAO group were reduced (Figures 3D,E). In contrast, the TSTT administration significantly restored the number of neurons (Figures 3D,E). From these data, we can conclude that TSTT promoted neuron survival and prevented PSCI-induced neuronal loss in the CA1 region.

### 3.4 TSTT ameliorated cognitive dysfunction in PSCI rats through suppressing neuronal apoptosis

**FIGURE 4:**
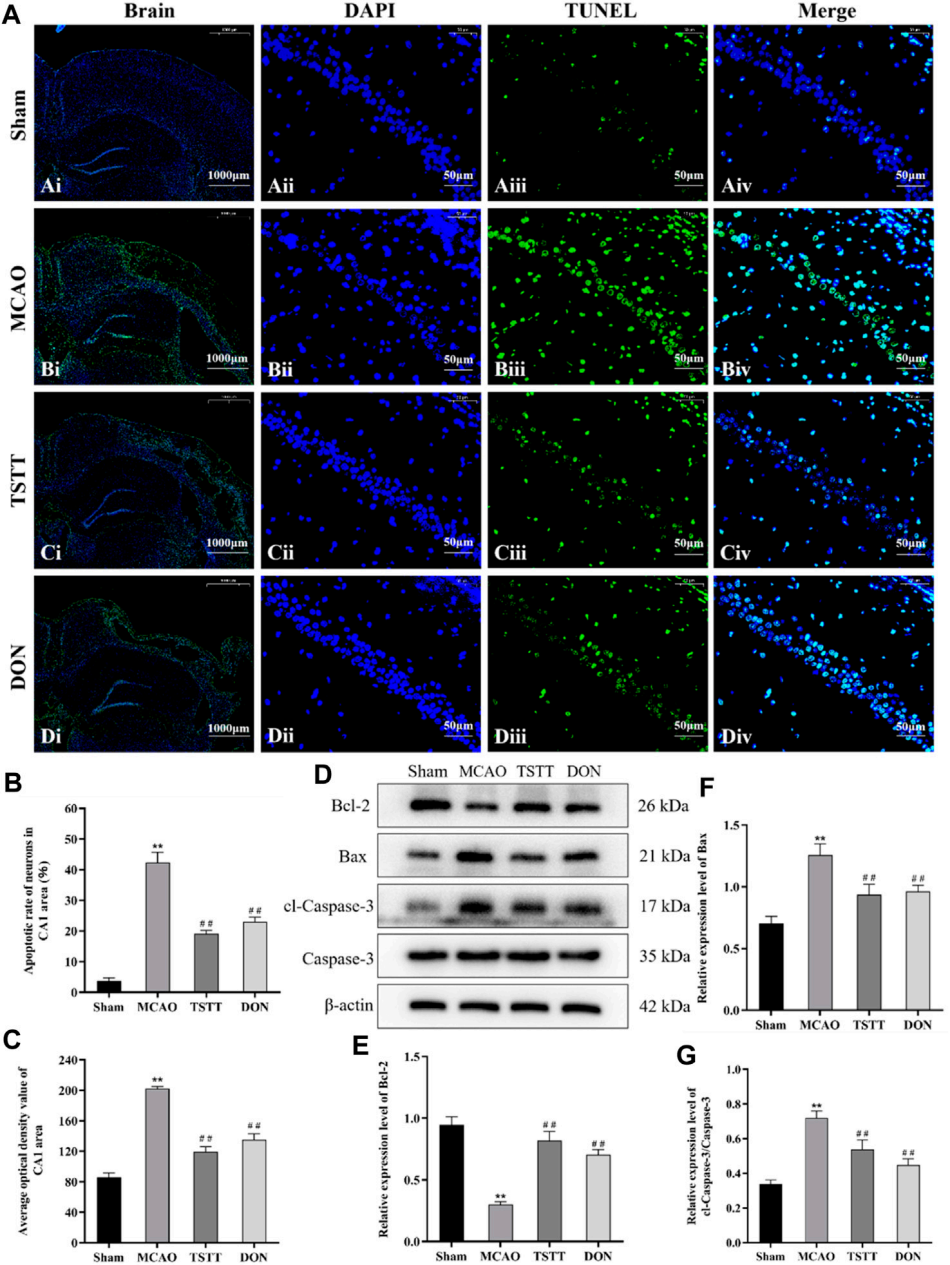
TUNEL staining and Bcl-2, Bax, cl-caspase-3, and caspase-3 proteins in the hippocampal CA1 region of rats from each group: **(A)** TUNEL staining of brain tissue: scale bar = 1,000 μm; DAPI/TUNEL/Merge: scale bar = 50 μm. **(B)** Neuronal apoptosis rate in the hippocampal CA1 region of rats in each group. **(C)** Average optical density of the hippocampal CA1 region in each group. **(D)** Expression of Bcl-2, Bax, cl-caspase-3, and caspase-3 proteins. **(E)** Relative expression of Bcl-2. **(F)** Relative expression of Bax. **(G)** Relative expression of cl-caspase-3/caspase-3. ^**^ *p* < 0.01 vs. Sham group; ^##^ *p* < 0.01 vs. MCAO group; *n* = 3.

We performed TUNEL staining to observe neuronal apoptosis and Western blot to evaluate the expression of pro-apoptotic proteins cl-caspase-3, caspase-3, Bax, and anti-apoptotic protein Bcl-2. The results showed different degrees of apoptosis in all groups except for a few apoptotic cells in rats from the Sham group. In the MCAO group, large apoptotic areas were observed in the cortex and ischemic penumbra. Compared with the Sham group, the apoptotic rates and average optical densities of neurons in rats from the MCAO group were significantly increased (*p* < 0.01). Compared with the MCAO group, the apoptotic rates and average optical densities of neurons in rats from the TSTT and DON groups were significantly decreased (*p* < 0.01), suggesting that ischemia can induce neuronal apoptosis in brain tissue, and TSTT treatment can suppress neuronal apoptosis. Additionally, compared with the sham group, the expression of the Bcl-2 protein in the hippocampus of rats from the MCAO group was significantly decreased, and the expression of Bax, and cl-caspase-3/caspase-3 proteins was significantly increased (*p* < 0.01). Compared with the MCAO group, the expression of the Bcl-2 protein in the hippocampus of rats in the TSTT and DON groups was significantly increased, and the expression of Bax and cl-caspase-3/caspase-3 proteins was significantly decreased (*p* < 0.01), suggesting that TSTT has an anti-apoptotic effect in rats with PSCI (Figure 4).

### 3.5 TSTT promoted neuronal synaptic remodeling in PSCI rats

**FIGURE 5:**
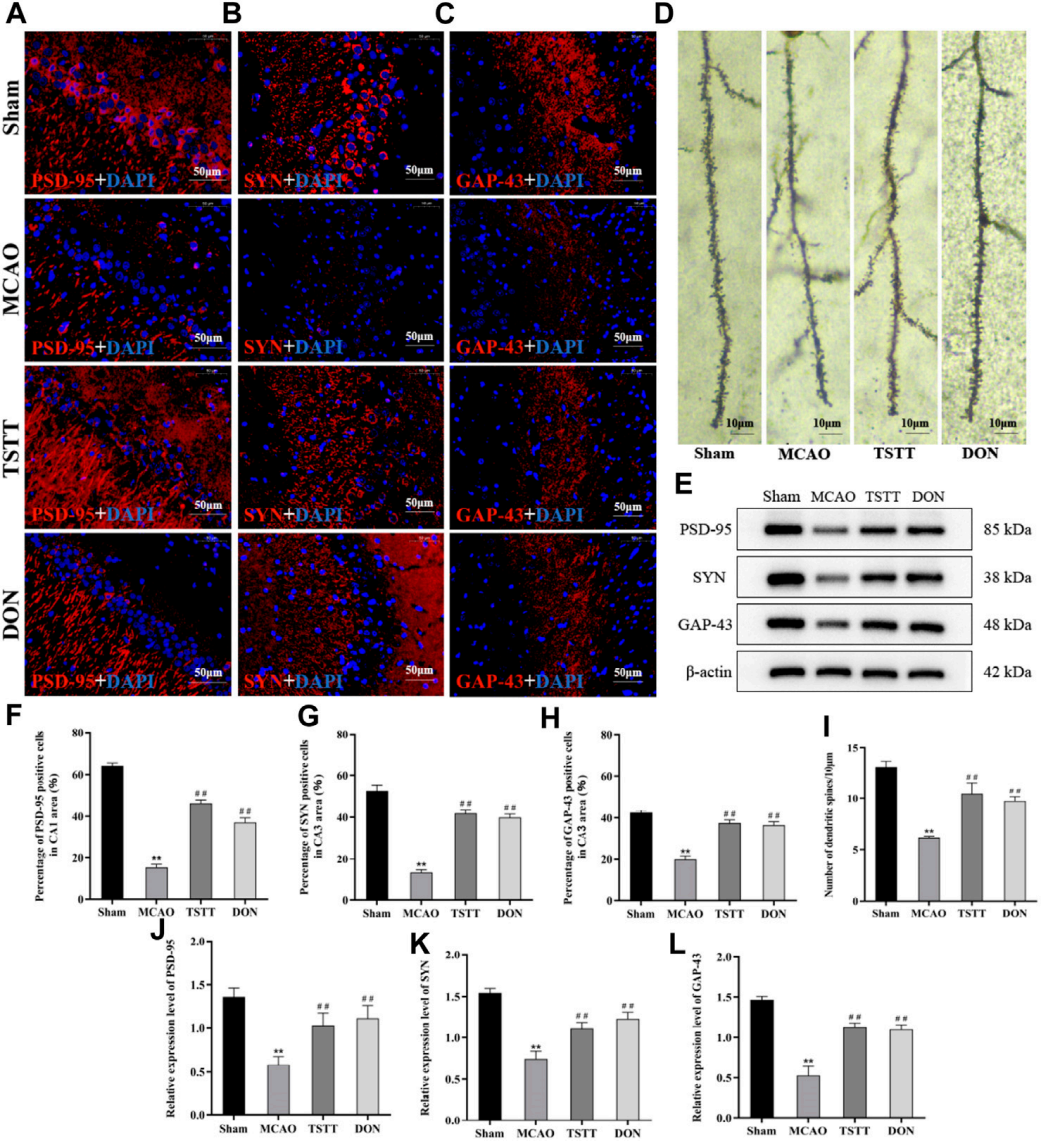
TSTT promoted synaptic remodeling in hippocampal neurons of PSCI rats. **(A)** PSD-95 immunofluorescence labeling in the hippocampal CA1 region; scale bar = 50 μm. **(B)** SYN immunofluorescence labeling in the hippocampal CA3 region; scale bar = 50 μm. **(C)** GAP-43 immunofluorescence labeling in the hippocampal CA3 region; scale bar = 50 μm. **(D)** Golgi staining of rats in each group; scale bar = 10 μm. **(E)** PSD-95, SYN, and GAP-43 protein expression in the hippocampus. **(F)** Population of PSD-95-positive cells. **(G)** Population of SYN-positive cells. **(H)** Population of GAP-43-positive cells. **(I)** Density of dendritic spines. **(J)** Relative expression of PSD-95. **(K)** Relative expression of SYN. **(L)** Relative expression of GAP-43. ^**^ *p* < 0.01 vs. Sham group; ^##^ *p* < 0.01 vs. MCAO group; *n* = 3.

To investigate whether TSTT can protect cognitive function of PSCI rats by promoting the remodeling of neuronal synapses, we observed the changes in dendritic spine density by Golgi staining. The results showed that the density of dendritic spines in the hippocampal CA1 region of rats from the MCAO group was significantly lower than that of the Sham group (*p* < 0.01). Compared with the MCAO group, the density of dendritic spines in the hippocampal CA1 region of rats from the TSTT and DON groups was significantly increased (*p* < 0.01), indicating that TSTT can increase the density of dendritic spines in hippocampal neurons of PSCI rats (Figure 5D).

Next, we also detected the expression and distribution of proteins associated with the synapse. Compared with the Sham group, the expression of PSD-95 (Figure 5A) in the hippocampal CA1 region and that of SYN and GAP-43 (Figures 5B,C) in the hippocampal CA3 region of rats from the MCAO group were significantly decreased (*p* < 0.01). Compared with the MCAO group, the expression of PSD-95 in the CA1 area and that of SYN and GAP-43 positive cells in the CA3 region of rats from the TSTT and DON groups were significantly increased (*p* < 0.01), suggesting that ischemia leads to a decrease in the expression of PSD-95 in the hippocampal CA1 region and that of SYN and GAP-43 in the CA3 region, and the expression of synaptic-related proteins can be upregulated after TSTT treatment.

The expression of PSD-95, SYN, and GAP-43 proteins in the hippocampus was detected by Western blot. The results showed that compared with the Sham group, the expression of PSD-95, SYN, and GAP-43 proteins in the hippocampus of the rats from the MCAO group was significantly decreased (*p* < 0.01). Compared with the MCAO group, the expression of PSD-95, SYN, and GAP-43 proteins in the hippocampus of the rats from the TSTT and DON groups increased significantly (*p* < 0.01). Taken together, TSTT can improve cognitive dysfunction in PSCI rats by increasing the dendritic spine density and promoting the expression of synapse-associated proteins (Figure 5E).

### 3.6 TSTT enhanced learning and memory capacity in rats with PSCI by activating Shh signaling

**FIGURE 6:**
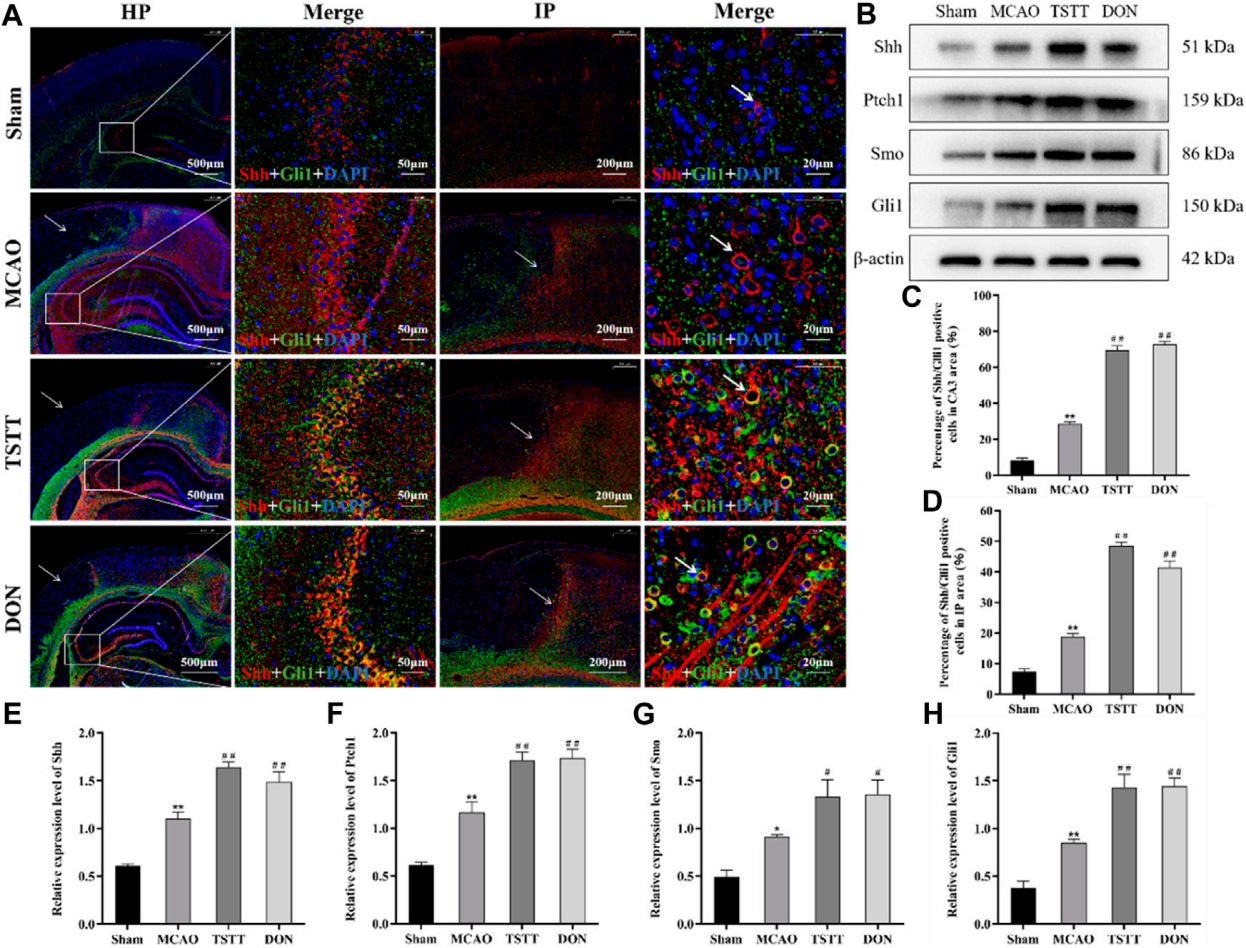
TSTT promoted Shh/Gli1 co-localization in the hippocampal CA3 region and ischemic penumbra in PSCI rats. **(A)** Shh/Gli1 co-localization staining in the hippocampal CA3 region and ischemic penumbra, HP: scale bar = 500 μm; HP-Merge: scale bar = 50 μm; IP: scale bar = 200 μm; IP-Merge: 20 μm. **(B)** Expression of Shh, Ptch1, Smo, and Gli1 proteins in the hippocampus. **(C)** Population of Shh/Gli1-positive co-localization positive cells in the hippocampal CA3 area. **(D)** Population of Shh/Gli1-positive co-localization cells in the hippocampal IP area. **(E)** Relative expression of Shh. **(F)** Relative expression of Ptch1. **(G)** Relative expression of Smo. **(H)** Relative expression of Gli1. ^*^ *p* < 0.05 or ^**^ *p* < 0.01 vs. Sham group; ^#^ *p* < 0.05 or ^##^ *p* < 0.01 vs. MCAO group; *n* = 3.

The results of Shh/Gli1 co-localization in the hippocampal CA3 region showed that the co-localization of Shh/Gli1 in rats from the Sham group was extremely low, showing only partial expression of Shh and Gli1, as shown in red labels. The co-localization of Shh/Gli1 in rats from the MCAO group was enhanced, as shown in purple red labels. The co-localization of Shh/Gli1 in rats from TSTT and DON groups is shown in orange labels. Compared with the Sham group, the number of Shh/Gli1-positive co-localization cells in the hippocampal CA3 region of rats from the MCAO group was significantly increased (*p* < 0.01). Compared with the MCAO group, the number of Shh/Gli1-positive co-localization cells in the hippocampal CA3 area of rats from the TSTT and DON groups was significantly increased (*p* < 0.01) (Figure 6A).

The results of Shh/Gli1 co-localization in the ischemic penumbra (IP) showed no damage in the cortical area of rats from the Sham group. The same position was selected for comparison. After modeling, only a few blue marker nuclei were observed in the ischemic cortical area of rats from each group. There were different degrees of Shh/Gli1-positive co-localization cells in the ischemic penumbra. Compared with the Sham group, Shh/Gli1-positive co-localization cells were significantly increased in the hippocampal IP area of rats from the MCAO group (*p* < 0.01). Compared with the MCAO group, the number of Shh/Gli1-positive co-localized cells in the IP area of rats from the TSTT and DON groups was significantly increased (*p* < 0.01) (Figure 6A).

The expression of Shh, Ptch1, Smo, and Gli1 proteins in the hippocampus was evaluated by Western blot. The results showed that compared with the Sham group, the expression of Shh, Ptch1, Smo, and Gli1 proteins in the hippocampus of rats from the MCAO group was significantly increased (*p* < 0.05 or *p* < 0.01). Compared with the MCAO group, the expression of Shh, Ptch1, Smo, and Gli1 proteins in the hippocampus of rats from the TSTT and DON groups was significantly increased (*p* < 0.05 or *p* < 0.01). Taken together, TSTT can protect PSCI rats by inhibiting apoptosis and promoting neuronal synaptic remodeling, with the activation of the Shh signaling pathway (Figure 6B).

## 4 Discussion

In this study, we examined the role of TSTT in PSCI by establishing an MCAO model. Our results suggest that spatial learning and memory are impaired in MCAO rats. This is attributed to excessive hippocampal neuronal apoptosis and downregulation of synapse-associated proteins. However, TSTT can rescue the cognitive dysfunction by inhibiting apoptosis and promoting neuronal synaptic remodeling by activating Shh.

The prevalence of PSCI ranges from 30% to 50% and often results in severe impairment of attention and executive function (Mellon et al., 2015). Unfortunately, there are still no conventional medications with convincing efficacy in the treatment of PSCI. Recently, there has been increasing evidence that traditional Chinese medicine may be able to promote cognitive function (Shen et al., 2022). Our previous study has reported that TSTT can enhance learning and memory capacity in rats with D-galactose-induced brain aging by inhibiting hippocampal neuron apoptosis. To verify whether TSTT could improve cognitive function for subjects with PSCI, the Morris water maze test was used to evaluate the spatial learning and memory capacity of MCAO rats after TSTT treatment (Wang et al., 2018).Our data have demonstrated that TSTT has a significantly ameliorative effect on cognitive dysfunction caused by acute cerebral ischemia. The hippocampus and cerebral cortex are important for the memory network in the brain. During the process of ischemic stroke, with the decrease in the blood flow, there will be severe brain injury, and excessive free radicals, calcium overload, and excitotoxicity can induce neuronal apoptosis, thereby resulting in cognitive decline or permanent memory impairment. In the present study, TSTT treatment significantly increased neuronal counts and Nissl bodies in the CA1 region of the hippocampus and cortex penumbra, indicating that TSTT intervention can protect brain damage of PSCI rats.

The major pathological changes in PSCI are the decrease in the synaptic number and connection density caused by acute injury (Lee et al., 2015), and the central nervous system exhibits considerable plasticity after injury, especially synaptic plasticity. The results showed that the density of dendritic spines, the number of positive cells, and the expression levels of PSD-95, SYN, and GAP-43 were significantly increased in MCAO rats treated with TSTT, indicating that TSTT intervention can enhance the synaptic plasticity of neurons in the hippocampus.

The Shh signaling pathway can influence the patterning of the nervous system, and it can also affect neurogenesis. In addition, it is involved in the formation and remodeling of hippocampal neural circuits, which are essential for learning and memory (Yao, Petralia, and Mattson, 2016). Previous studies have implicated the Shh pathway in the protective role of ischemic stroke by modulating the processes of apoptosis, glutamate excitotoxicity, neuroplasticity, intracranial angiogenic, neurogenic processes, and inflammation (Liu et al., 2018). Here, we show that the Shh pathway is more activated in TSTT than in MCAO, as evidenced by the enhanced expression of PSD-95, SYN, and GAP-43. We have confirmed that TSTT can stimulate various protective mechanisms through the activation of the Shh pathway to promote synaptic remodeling, suggesting a potential for TSTT intervention to target the Shh pathway, but the specific regulatory mechanism of TSTT on the Shh signaling pathway still needs further exploration.

Shh is an essential survival factor in various types of cells and can inhibit apoptosis (Charrier et al., 2001). Activation of the PI3K/Akt pathway by the exogenous Shh peptide protects astrocytes by inhibiting apoptosis induced by H_2_O_2_ (Yang C. et al., 2021). Another study showed that the inhibition of the Shh signal transduction by miR-30c led to a decrease in apoptosis rates and differentiation of P19 cells (Liu et al., 2016). In this study, it was found that TSTT treatment significantly downregulated the levels of select apoptosis-related proteins including Bax and cl-caspase-3 caspase-3 ratios, while upregulated the level of Bcl-2. Moreover, TSTT treatment reduced the TUNEL-positive cell number in the cerebral cortex of model rats. This implies that the anti-apoptotic effect of TSTT treatment is similar to that of the Shh peptide. The activation of the typical Shh pathway is responsible for the upregulation of the anti-apoptotic Bcl-2 gene by Gli-1 (Bigelow et al., 2004). Caspase-3 is an enzyme that triggers apoptosis. Shh can inhibit apoptosis by downregulating caspase-3 (Kumari et al., 2014). The aforementioned results indicate that TSTT restores cognitive function by activating the Shh signaling pathway and attenuating apoptosis, which is in line with previous findings.

Previous studies have shown that neurons can secrete SHH to promote synaptic remodeling. Furthermore, astrocytes can also secrete Shh, which interacts with neurons to increase the levels of genes expressed by astrocytes, including the Shh target gene Sparc, which promotes synaptic remodeling by increasing synapse formation and enhancing synaptic connectivity (Xie et al., 2022). Therefore, we hypothesize that excessive apoptosis of neuronal cells after stroke leads to a large loss of Ptch1, which activates the Shh signaling pathway in neurons in the ischemic penumbra and then targets the synaptic remodeling-related genes such as Sparc to promote synaptic remodeling. TSTT may exert neuroprotective effects by enhancing and amplifying Shh signaling to promote synaptic remodeling. Of course, we do not rule out the possibility that the Shh pathway may initiate a signaling cascade that is then mediated by other molecular pathways to activate synaptic remodeling.

The hippocampal network is critical for forming accurate memories and representing a spatial location, and the cooperation of attraction and repulsion is essential for the neuronal circuit assembly (Igarashi et al., 2014). Previous studies have shown that axons are guided by molecular cues that extend to the brain areas to which they are directed and eventually form synaptic connections with partner neurons (Xie and Harwell, 2021). Teneurin-3 (Ten3), expressed in the hippocampus, is a type II transmembrane protein that controls target selection from CA1 to subiculum axons (Berns et al., 2018). A subsequent study further confirmed that this targeting process is precisely regulated by Lphn2/Ten3 and Ten3/Ten3-mediated heterophilic repulsion and homophilic attraction, respectively (Pederick et al., 2021). Shh has been shown to exhibit axon guidance activity, which may act in concert with Netrin-1 as a chemoattractant for commissural axons (Seiradake, Jones, and Klein, 2016). As a key link in the rehabilitation process after stroke, Shh activation may stimulate axon extension, accelerating intercommunication with specific neurons and synapses (Yao, Petralia, and Mattson, 2016). Whether TSTT remodels the neural circuits of the hippocampus by controlling axon guidance through regulating Lphn2 and Ten3 levels is not clear. Therefore, we speculate that TSTT may regulate the axon repulsion and attraction mediated by Lphn2 and Ten3 through the Shh, which is crucial for axon guidance during the remodeling of hippocampal neural circuits after PSCI. This is a novel research direction in the future.

New research shows that by regulating axonal remodeling through the GSK-3/β-catenin/CRMP-2 pathway, short-term interventions with TTST may promote the recovery of brain function (Yang et al., 2021a). Another study examining the role of 30 days TTST gavage in the chronic phase of the ischemic stroke demonstrated that TTST improved gait impairment in MCAO rats by activating axonal remodeling and the PI3K/AKT/GSK-3/CRMP-2 pathway (Yang et al., 2021b). In the present study, we focused on the effects of long-term intervention with TSTT on learning and memory function in the chronic phase of ischemic stroke. We demonstrated that TTST promotes brain remodeling by promoting axonal remodeling. This may be mediated by the Shh signaling pathway. This is an addition to the study of the potential mechanisms by which TTM may promote remodeling of the brain.

## 5 Conclusion

Our study has provided strong evidence that TSTT can promote neurological recovery of PSCI rats. This is of great significance in providing a novel treatment strategy for patients with PSCI. In conclusion, TSTT can suppress apoptosis and enhance synaptic plasticity in the hippocampus in PSCI rats by activating the Shh signaling pathway, thereby alleviating cognitive dysfunction. This is a pioneering study for opening new horizons in treating PSCI and developing Chinese herbal medicine.

## Data Availability

The original contributions presented in the study are included in the article/Supplementary Material; further inquiries can be directed to the corresponding author.
